# Acute on Chronic Immune Thrombocytopenia as a Cause of Thrombocytopenia in Sepsis

**DOI:** 10.7759/cureus.8168

**Published:** 2020-05-17

**Authors:** Pujitha Kudaravalli, Venkata Satish Pendela, Harvir Singh S Gambhir

**Affiliations:** 1 Internal Medicine, State University of New York (SUNY) Upstate Medical University, Syracuse, USA; 2 Internal Medicine, Rochester General Hospital, Rochester, USA

**Keywords:** immune thrombocytopenia, sepsis, immunoglobulins, steroids

## Abstract

Immune thrombocytopenia (ITP) is a diagnosis of exclusion and can be challenging at times to make the diagnosis. We herein present a case of a 73-year-old male with a history of chronic ITP, who presented to the hospital with sepsis and developed thrombocytopenia. His thrombocytopenia did not improve with resolution of sepsis but improved with ITP treatment including immunoglobulins and steroids. Platelet-associated IgG antibody levels are inversely proportional to platelet counts. The antibody levels are increased in sepsis. We would like to highlight that other causes of thrombocytopenia should also be considered in sepsis.

## Introduction

Immune thrombocytopenia (ITP) is a diagnosis of exclusion, with an incidence of two per 100,000 adults with a median age of 50 [1]. Most adults have chronic ITP and the pathogenesis of ITP continues to remain unclear. We herein present a case of acute on chronic ITP in a patient with sepsis who responded to ITP treatment with steroids and intravenous immunoglobulin. We would like to highlight that acute on chronic ITP should be considered as a cause of thrombocytopenia in sepsis in patients with chronic ITP as there is also increased production of platelet-associated immunoglobulin G antibodies during sepsis.

## Case presentation

A 73-year-old male presented to the ED with fever and lethargy for one day. His past medical history is significant for hyperlipidemia, hypertension, gout, and chronic ITP. His past surgical history included a recent dental procedure with a dental implant placement.

**Figure 1:**
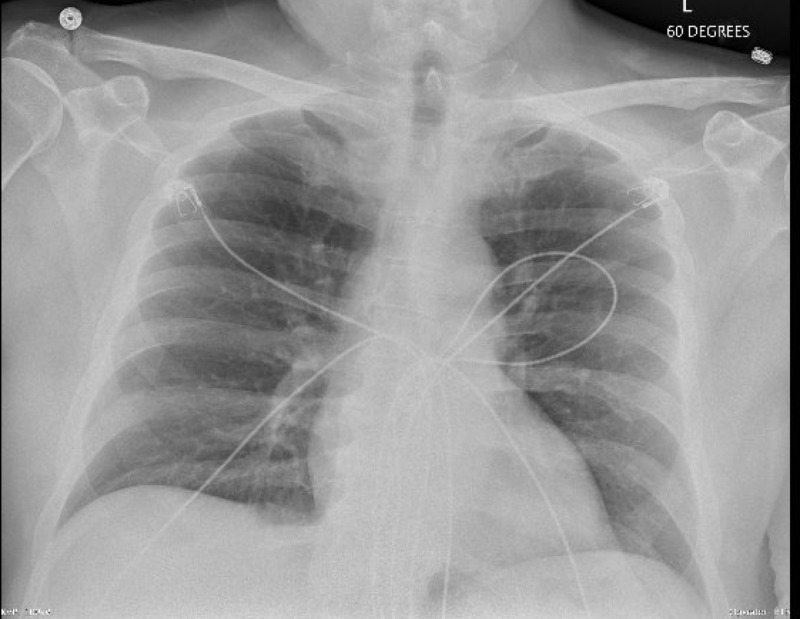
Chest X-ray. Posterior anterior view of the X-ray of the chest showing no features of acute infection

**Figure 2:**
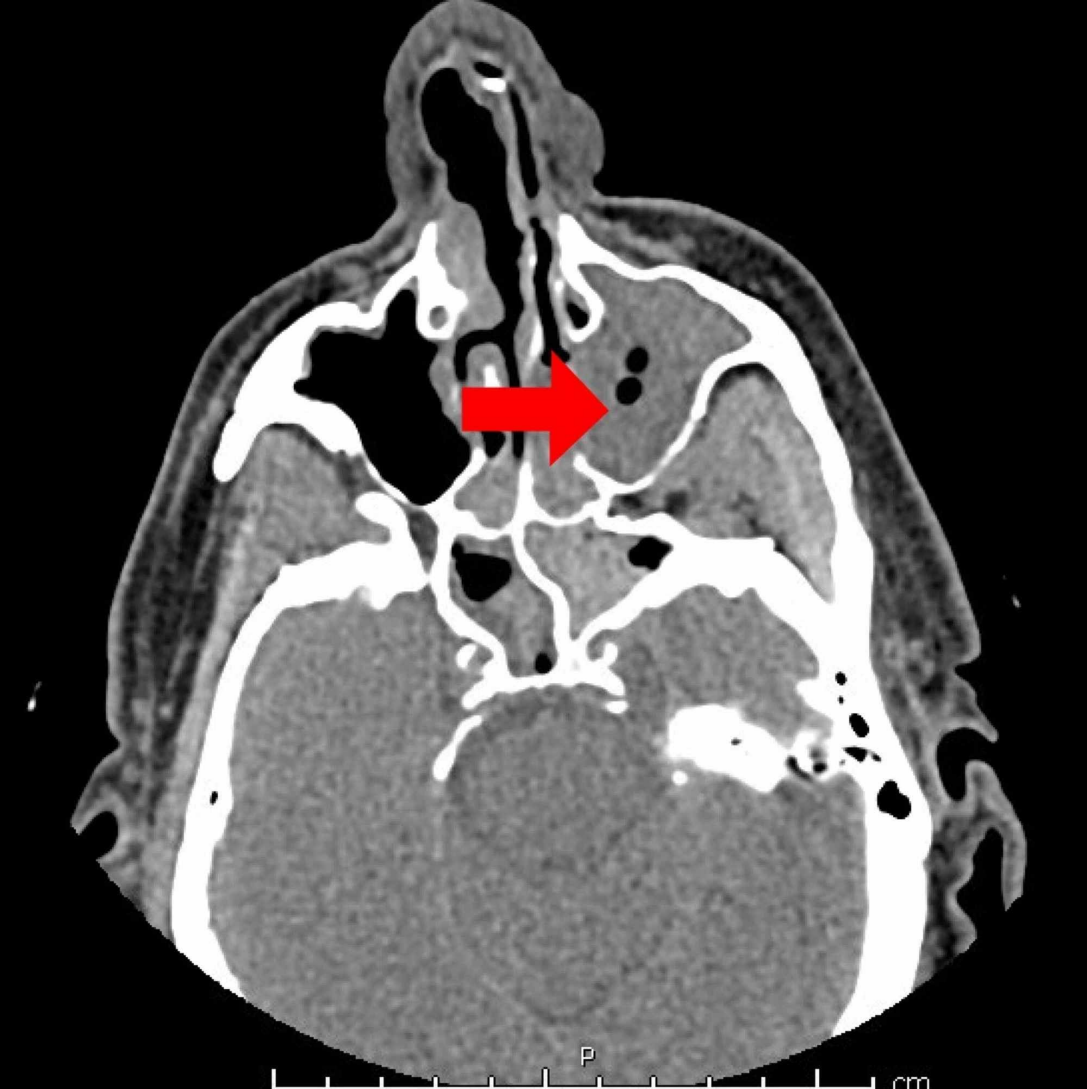
CT maxillofacial without contrast. CT scan maxillofacial without contrast showing severe opacification of left maxillary sinus as pointed by the red arrow

On examination, his temperature was 39.3 degree Celsius, blood pressure was 137/89 mmHg, heart rate was 99 beats/min. Cardiovascular system and respiratory system were unremarkable. Laboratory findings showed a white blood cell (WBC) count of 3600/mm^3^, hemoglobin of 12.2 g/dL with a hematocrit of 36.3%, and platelet count of 45000/mm^3^. The baseline platelet count of the patient was 90000/mm^3^. Chest X-ray (as shown in Figure 1), respiratory panel, and urinalysis were negative for infection. Blood cultures did not show any growth. CT maxillofacial without contrast showed metallic implant in the left maxillary tooth and moderate to severe paranasal sinusitis (as shown in Figure 2). The patient was diagnosed with sepsis secondary to transient bacteremia secondary to dental procedure versus sinusitis and was started on broad-spectrum antibiotics.

The patient improved clinically with antibiotics and the signs of active infection resolved. However, his platelet count continued to worsen with a nadir of 25000/mm^3^. Hematology was consulted and the peripheral blood smear was reviewed which was unremarkable with no hemolysis. Other causes of thrombocytopenia such as infection with HIV, hepatitis C, autoimmune panel, thyroid stimulating hormone (TSH) were ruled out. Of note, the patient had a bone marrow biopsy nine months before presentation which showed 5.6% plasma cells without evidence of clonality and high-normal number of megakaryocytes with thrombocytopenia consistent with peripheral destruction or sequestration confirming his previous diagnosis of chronic ITP. A presumptive diagnosis of acute on chronic ITP was made. He was started on IV immunoglobulins 1 mg/kg of ideal body weight before considering an immunosuppressant in a patient with infection and there was an improvement in platelet counts from 25000/mm^3^ to 41000/mm^3^. He was subsequently started on prednisone 100 mg and his platelets drastically improved to 120000/mm^3^. He was discharged on prednisone 100 mg PO daily for 7 days with an outpatient hematology follow up.

## Discussion

Platelets are an integral part of the immune response, inflammation, pathogen killing, and tissue repair in sepsis in addition to hemostasis and thrombosis. Thrombocytopenia is commonly seen in sepsis and is also an important marker of prognosis in sick ICU patients. In a study done by Venkata et al., it was found that 47.6% of patients with sepsis had thrombocytopenia. Development of thrombocytopenia in sepsis is attributed to consumption from sequestration and destruction, immune-mediated mechanisms secondary to nonspecific platelet-associated antibodies, and cytokine-driven hemophagocytosis of platelets [2-3]. Thrombocytopenia in sepsis is also caused by decreased platelet production in the bone marrow caused by inhibitory effects of toxins, drugs, or inflammatory mediators.

Persistent thrombocytopenia is associated with increased mortality and longer ICU stay [2, 4]. It is also observed that patients with thrombocytopenia are more sick [5]. The most common practice of management for thrombocytopenia in sepsis is treatment of underlying infection and continued monitoring but it is important to recognize that in a patient with chronic ITP, thrombocytopenia can be secondary to an exacerbation of underlying ITP. Immune system may be involved in the reduction in platelet count in septicemia. In a study done by Matschke et al., it was shown that platelet associated IgG antibodies were elevated in septic patients and their levels were inversely proportional to the platelet counts [6].

Immune thrombocytopenia can be primary or secondary, acute (less than six months) or chronic. Secondary forms of the disease are thrombotic thrombocytopenic purpura, hemolytic uremic syndrome, disseminated intravascular coagulation, paroxysmal nocturnal hemoglobinuria, infection with hepatitis C and HIV, drugs like heparin and quinidine, lymphoproliferative disorders like chronic lymphocytic leukemia. A patient is diagnosed with ITP after the above differentials are ruled out. It is hypothesized that there is development of immunoglobulin G antibodies targeting platelet membrane glycoprotein IIb-IIIa. These platelets that are coated with IgG antibodies undergo accelerated destruction in the liver/spleen or intramedullary destruction of antibody coated platelet by macrophages [4, 7].

The first line of treatment for ITP is prednisone 1 mg/kg for two to four weeks. Repeated cycles of pulsed high dose dexamethasone have also shown similar results as that of daily prednisone therapy [8]. Splenectomy was a popular treatment for ITP but recent data show that <20% of patients now undergo splenectomy for ITP. Anti-D immunoglobulin therapy has also demonstrated efficacy in rapidly increasing platelet counts in patients with an intact spleen and it used to delay splenectomy in adults. It has an advantage over intravenous immunoglobulin (IVIG) with regard to expense and longer duration of action [9]. Rituximab, thrombopoietin receptor agonist (TPO-RA), and IVIG are other treatment modalities. Several novel therapies are on the horizon for ITP including antibodies targeting CD40-CD154 interaction between B and T cells, novel agents increasing platelet production, including amifostine, treatments targeting the Fc receptor [10].

## Conclusions

We would like to highlight that acute on chronic ITP should be considered as a cause of thrombocytopenia in patients with chronic ITP when they are admitted to the hospital with sepsis. Not all thrombocytopenia in patients can be related to sepsis and other causes should also be considered. The treatment of ITP is different from that of sepsis-induced thrombocytopenia and physicians should be vigilant of this condition. 
